# The First Detection of Equine Coronavirus in Adult Horses and Foals in Ireland

**DOI:** 10.3390/v11100946

**Published:** 2019-10-14

**Authors:** Manabu Nemoto, Warren Schofield, Ann Cullinane

**Affiliations:** 1 Virology Unit, The Irish Equine Centre, Johnstown, Naas, Co. Kildare W91 RH93, Ireland; nemoto_manabu@equinst.go.jp; 2 Equine Research Institute, Japan Racing Association, Shimotsuke, Tochigi 329-0412, Japan; 3 Troytown Grey Abbey Equine Hospital, Green Road, Co. Kildare R51 YV04, Ireland; warrenschofield@gmail.com

**Keywords:** equine coronavirus, Ireland, enteric disease

## Abstract

The objective of this study was to investigate the presence of equine coronavirus (ECoV) in clinical samples submitted to a diagnostic laboratory in Ireland. A total of 424 clinical samples were examined from equids with enteric disease in 24 Irish counties between 2011 and 2015. A real-time reverse transcription polymerase chain reaction was used to detect ECoV RNA. Nucleocapsid, spike and the region from the p4.7 to p12.7 genes of positive samples were sequenced, and sequence and phylogenetic analyses were conducted. Five samples (1.2%) collected in 2011 and 2013 tested positive for ECoV. Positive samples were collected from adult horses, Thoroughbred foals and a donkey foal. Sequence and/or phylogenetic analysis showed that nucleocapsid, spike and p12.7 genes were highly conserved and were closely related to ECoVs identified in other countries. In contrast, the region from p4.7 and the non-coding region following the p4.7 gene had deletions or insertions. The differences in the p4.7 region between the Irish ECoVs and other ECoVs indicated that the Irish viruses were distinguishable from those circulating in other countries. This is the first report of ECoV detected in both foals and adult horses in Ireland.

## 1. Introduction

Equine coronavirus (ECoV) is a positive-stranded RNA virus and belongs to the species *Betacoronavirus 1* in the genus *Betacoronavirus* [1,2]. The clinical signs associated with ECoV infection during outbreaks in the USA [3] and Japan [4,5,6] were fever, anorexia, lethargy and diarrhoea. The same clinical signs were also recorded in an experimental challenge study using Japanese draft horses [7]. The main transmission route is considered to be faecal–oral [7] and ECoV is usually detected in faecal samples. However, the molecular detection of ECoV in faeces from horses with diarrhoea, does not prove causation. Coronaviruses can cause both enteric and respiratory disease in many avian and mammalian species but ECoV is less likely to be found in respiratory secretions than in faeces [8,9]. 

Both molecular and seroepidemiology studies suggest that ECoV may be more prevalent in the USA than in other countries [10]. ECoV was detected in samples collected from equids in 48 states of the USA [11]. In central Kentucky, approximately 30% of both healthy and diarrheic Thoroughbred foals were infected with ECoV [12]. All of the qPCR positive foals with diarrhoea were co-infected with other pathogens such as rotavirus or *Clostridium perfringens*, suggesting that there was potential for ECoV to be over-diagnosed as a causative agent in complex diseases. In contrast in Japan, although an outbreak of diarrhoea occurred among ECoV-infected draft horses at one racecourse [4,5,6], there have been no similar outbreaks subsequently, and all rectal swabs collected from diarrheic Thoroughbred foals were negative. Furthermore, only 2.5% of the rectal swabs collected from healthy foals in the largest Thoroughbred horse breeding region in Japan were positive for ECoV [13]. In France, 2.8% of 395 faecal samples and 0.5% of 200 respiratory samples collected in 58 counties tested positive for ECoV [9]. Similar to the reports from Japan and France, a low prevalence of ECoV was also observed in the UK [14], Saudi Arabia and Oman [15]. The objective of this study was to investigate the presence of ECoV in clinical samples submitted to a diagnostic laboratory in Ireland. The samples were tested by real-time reverse transcription polymerase chain reaction (rRT-PCR) as it has been shown to be the most sensitive diagnostic method for ECoV [16] and is routinely employed as an alternative to virus isolation in diagnostic laboratories worldwide, both for timely diagnosis and in epidemiological studies [9,10]. Virus isolation and biological characterisation were beyond the capacity of this study, which was similar in scope to that of the studies in horse populations in the USA, Europe and Asia [8,9,13,14].

## 2. Materials and Methods 

Faecal samples and rectal swabs collected from equids with enteric disease were included in this study. Samples were collected in 24 Irish counties between 2011 and 2015. All samples (65 in 2011, 69 in 2012, 97 in 2013, 97 in 2014 and 96 in 2015) were stored at −80 °C prior to testing by rRT-PCR. Faecal samples were diluted 1:10 in phosphate buffered saline (PBS), and rectal swabs were immersed in PBS. The suspensions were clarified by centrifugation at 2000 × g for 10 min, and viral RNA was extracted from the supernatant using the LSI MagVet Universal Isolation Kit (Thermo Fisher Scientific, Cambridge, MA, USA).

The rRT-PCR assay was performed as previously described using a primer set targeting the nucleocapsid (N) gene (ECoV-380f, ECoV-522r and ECoV-436p) [3] (Table 1) and AgPath-ID One-Step RT-PCR Kit (Thermo Fisher Scientific, MA, USA) according to the manufacturer’s instructions. To prove that the extraction was successful and that there was no inhibition during rRT-PCR amplification, an internal positive control primer/probe (PrimerDesign, Southampton, UK) was added to the master mix. Thermal cycling conditions were; 48 °C for 10 min and 95 °C for 10 min, followed by 40 cycles at 94 °C for 15 s and 60 °C for 45 s. 

The SuperScript III One-Step RT-PCR System with Platinum Taq High Fidelity (Thermo Fisher Scientific, MA, USA) was used for sequencing analysis of two of the five ECoV samples identified. There was inadequate viral nucleic acid in the other three samples for sequencing. The primer sets used to amplify the nucleocapsid (N) gene [4], the partial spike (S) gene [9], and the region from the p4.7 to p12.7 genes of non-structural proteins (Oue, personal communication) are shown in Table 1. The RT-PCR products were sequenced commercially by GATC Biotech (Cologne, Germany). Sequence analysis was performed using the BLAST and CLUSTALW programs, and Vector NTI Advance 11.5 software (Thermo Fisher Scientific, MA, USA). Phylogenetic analysis of nucleotide sequences was conducted with MEGA software Version 5.2 [17]. A phylogenetic tree was constructed based on nucleotide sequences of the K2+G (N gene) and TN93 (S gene) using the maximum likelihood method. MEGA software was used to select the optimal substitution models. Statistical analysis of the tree was performed with the bootstrap test (1000 replicates) for multiple alignments. The complete genome sequences of NC99 (EF446615) [2], Tokachi09 (LC061272), Obihiro12-1 (LC061273) and Obihiro12-2 (LC061274) [1], the N (AB671298) and S (AB671299) genes of Obihiro2004, the N gene of Hidaka-No.61/2012 (LC054263) and Hidaka-No.119/2012 (LC054264) [13], the S gene of ECoV_FRA_2011/1 (KC178705), ECoV_FRA_2011/2 (KC178704), ECoV_FRA_2012/1 (KC178703), ECoV_FRA_2012/2 (KC178702) and ECoV_FRA_2012/3 (KC178701) [9] were used in sequence and/or phylogenetic analysis.

The accession numbers registered in GenBank/EMBL/DDBJ are as follows: the complete sequences of the N gene; 11V11708/IRL (LC149485) and 13V08313/IRL (LC149486), the partial sequences of the S gene; 11V11708/IRL (LC149487) and13V08313/IRL (LC149488) and the complete sequences from the p4.7 to p12.7 genes; 11V11708/IRL (LC149489) and13V08313/IRL (LC149490). 

## 3. Results

Five samples (11V11708/IRL/2011 and 11V06761/IRL/2011 collected in 2011, and 13V08313/IRL/2013, 13V08314/IRL/2013 and 13V07530/IRL/2013 collected in 2013) tested positive for ECoV. All samples collected in 2012, 2014 and 2015 tested negative. Sample 11V11708/IRL/2011 was collected in November 2011 from a donkey foal in County Cork. Samples 13V08313/IRL/2013 and 13V08314/IRL/2013 were collected on the same day in April 2013 from adult horses on a farm in County Clare. At the time of sample collection, both horses were described as having mild diarrhoea for 24 to 48 h. This resolved within a week. Samples 11V06761/IRL/2011 and 13V07530/IRL/2013 were collected in County Kildare from diarrheic foals on two separate premises in March 2011 and April 2013, respectively. One six-week-old foal was the only clinical case on a public Thoroughbred stud farm with approximately 30 mares when it presented with diarrhoea. Recovery took over three weeks during which it received fluid therapy, probiotics, antiulcer medication and antibiotics. The second foal was a 14-day-old filly, which had been hospitalised with diarrhoea two days prior to sample collection. The foal responded well to supportive treatment and at the time of sample collection, the diarrhoea had resolved. The five ECoV positive samples tested negative for equine rotavirus.

The nucleotide sequences of the complete N gene, the partial S gene and the region from the p4.7 to p12.7 genes of two positive samples (11V11708/IRL/2011 and 13V08313/IRL/2013) were determined. The nucleotide identities of the N and S genes of the two Irish ECoVs were 99.8% (1338/1341 nucleotides) and 99.5% (650/653 nucleotides), respectively. The nucleotide identities of the N gene of the two Irish ECoVs and the ECoVs from other continents are summarised in Table 2.

**Figure 1:**
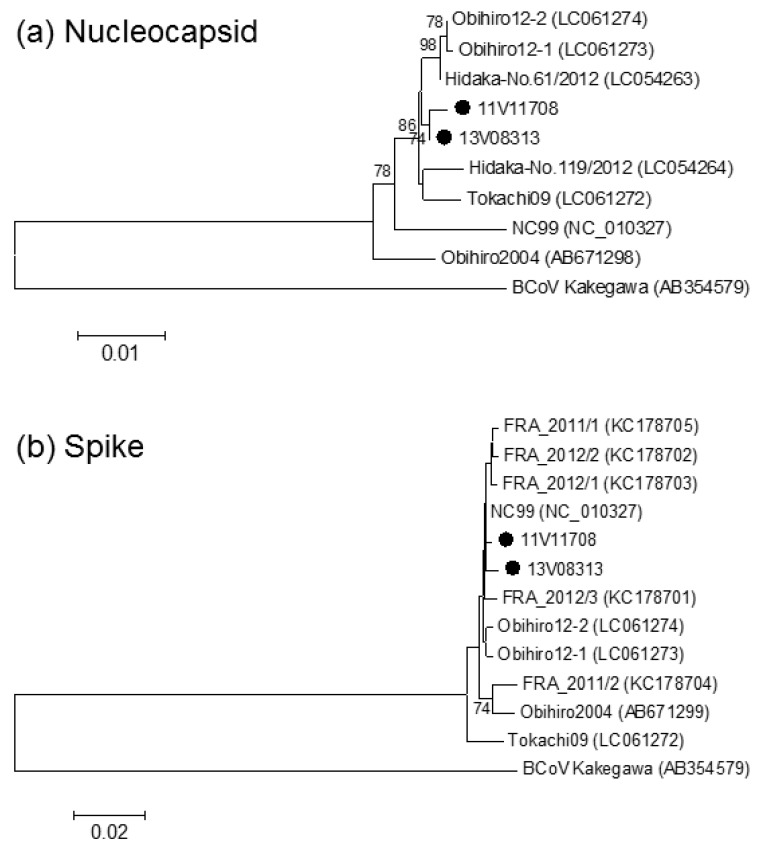
Phylogenetic analysis of the nucleotide sequences of the complete nucleocapsid (**a**) and the partial spike (**b**) genes of equine coronavirus. Closed circles indicate two of the equine coronaviruses detected in Ireland (11V011708 and 13V03813). The percentage bootstrap support is indicated by the value at each node; values <70% are omitted. Scale bars indicate nucleotide substitutions per site. BCoV (Bovine coronavirus) Kakegawa strain is used as an out-group.

Phylogenetic analysis was performed for the nucleotide sequences of the complete N and partial S genes (Figure 1). The analysis for the N gene showed that Irish ECoVs were independently clustered although they were closely related to Japanese viruses identified after 2009. In the phylogenetic tree of the S gene, Irish ECoVs were closely related to all other ECoVs analysed.

The length of the region from the p4.7 to p12.7 genes in the two viruses was 544 base pairs. Compared with NC99, Irish ECoVs, had a total of 37 nucleotide deletions within p4.7 and the non-coding region following the p4.7 gene. Compared with Obihiro 12-1 and 12-2, Irish ECoVs had a three-nucleotide insertion. When compared with Tokachi09, the Irish ECoVs had a 148-nucleotide insertion (see Figure S1). The p12.7 gene of the two Irish ECoVs did not have deletions or insertions, and the nucleotide identities were 98.8–99.7% between these viruses and the other ECoVs (NC99, Tokachi09, Obihiro12-1 and Obihiro12-2).

## 4. Discussion

This study provides the first report of ECoV circulating in Ireland, the third European country with a significant horse industry where the virus has been detected in horses with enteric disease. However, detection of ECoV in faeces samples from horses with enteric disease does not prove causation. In this study, 424 samples collected between 2011 and 2015 from equids with enteric disease were tested, and only five samples (1.2%) were positive for ECoV. The inclusion of an internal positive control in the rRT-PCR eliminated the possibility of false negative results due to the presence of PCR inhibitors but the high content of nucleases associated with faeces samples may have caused some RNA degradation. However, this low prevalence of ECoV is similar to that identified in France [9] and among Thoroughbred foals in Japan [13].

Although ECoV has been identified on three continents, little is known about the genetic and pathogenic diversity in field viruses. In this study, sequence and phylogenetic analysis (Figure 1) demonstrated a high level of homology between viruses detected in a donkey and a horse in two provinces in Ireland in different years. This suggests that Irish ECoVs may have low genetic diversity. Compared with the ECoVs of other countries, the N, S and p12.7 genes of the two Irish viruses were highly conserved. In contrast, the region from p4.7 and the non-coding region following the p4.7 gene had deletions or insertions (Figure S1). Because of polymorphism in this region, this region could be useful for epidemiological investigation [5]. The differences in the p4.7 region between the Irish ECoVs and other ECoVs indicated that the viruses in Ireland may be distinguishable from those circulating in other countries. The positive samples were collected in November (1), March (1) and April (3) in this study. Higher case numbers are identified in the USA during the colder months (October to April) [11], and our results were consistent with the circulation period in USA. It has been reported that outbreaks mainly occurred among adult riding, racing and show horses in USA [11]. The choice of cases to include in the current study may not have been optimal for detection of ECoV as the majority of samples were from foals. However, two positive samples were collected from adult horses in a combined riding school/show jumping yard in the West of Ireland. At the time of sample collection in April 2013, the monthly mean temperatures were below long-term average and in parts of the West, were the coldest in 24 years [18]. Cold weather may have been a predisposing factor to the ECoV infection on the farm. 

Two positive samples were collected from Thoroughbred foals. A faeces sample collected from one foal with severe watery diarrhoea and inappetance was positive for ECoV but a sample collected three days later tested negative. A potential difficulty in detecting ECoV from naturally infected horses has been noted previously as serial samples from seven sick horses in the USA suggested that ECoV only persisted for three to nine days in faeces [3]. In both cases, the diarrhoea may have been caused by other unidentified coinfecting pathogens as has been suggested by investigators in the USA [12].

This is the first report of ECoV detection in faeces samples from both foals and adult horses in Ireland. The viruses identified in Ireland are genetically closely related to the Japanese viruses and the results of this study give no indication of significant genetic or phenotypic diversity. In recent years, there has been an increase in awareness and testing for ECoV in the USA and elsewhere [10]. Horse breeding and racing activities in Ireland are the most prominent and important of any country on a per capita basis. There are over 50 Thoroughbred horses per 10,000 of population in Ireland, compared to between three and five for Great Britain, France and the USA [19]. Thus, an investigation of ECoV in Ireland is pertinent not only to increase awareness nationally of the epidemiology of the virus and promote discussion on its clinical importance, but also to inform the industry globally of the health status of Irish horses. Ireland exports horses all over the world. By illustration, in 2016 the country was the second biggest seller of bloodstock at public auctions second only to the USA [19]. 

Many questions remain with regard to the clinical significance of ECoV. The outbreak at a draft-horse racetrack in Japan in 2009 affected 132 of approximately 600 horses and resulted in non-starters and the implementation of movement restrictions [4]. However, draft horses appear to have a higher infection rate than other breeds and an outbreak of similar severity has not been reported in Thoroughbred racehorses [10,20]. The much higher incidence of ECoV positive Thoroughbred foals identified in Kentucky compared to similar populations internationally suggests an increased susceptibility to ECoV infection in that population. In the past, specific environmental factors were associated with extensive reproductive loss in the Kentucky area and to a lesser extent in other states [21], but predisposing regional factors such as differences in management, environment or husbandry have not been identified for ECoV. It has been suggested that ECoV is a coinfecting agent in foals with diarrhoea and clinical infections have predominantly been reported in adult horses with a mono-infection with EcoV [10]. There was no indication from the results of this study that coronavirus is a major cause of diarrhoea in Irish horses but the introduction of rRT-PCR as a routine diagnostic test will assist in elucidating the significance of this virus to the Irish breeding, racing and sports industries. The primary focus in future will be on testing adult horses that present with anorexia, lethargy, fever and changes in faecal character as a significant association has been demonstrated between this clinical status and molecular detection of ECoV in faeces [11].

## Figures and Tables

**Table 1:** Primers and probe for rRT-PCR and sequencing primers.

Primer Name	Sequence 5′-3′	Use	Target	Reference
ECoV-380-F	TGGGAACAGGCCCGC	PCR	Nucleocapsid	[3]
ECoV-522-R	CCTAGTCGGAATAGCCTCATCAC			
ECoV-436-probe	TGGGTCGCTAACAAG			
ECoV-N-F	TCAGGCATGGACACCGCATTGTT	Sequencing	Nucleocapsid	[4]
ECoV-N-R	CCAGGTGCCGACATAAGGTTCAT			
ECoV-S1-F	CAATGCCTTTATGGCTTGGT	Sequencing	Spike Gene	[9]
ECoVS1-R	AAACTCGGAAGGGATCTGAA			
ECoV-p4.7-F	TAATCGGCCTTGCTGGTGTAGC	Sequencing	p4.7 to p12.7 genes	Oue (personal communication)
ECoV-p4.7-R	GCTTCATCAGCAGTCCAGGTA			

**Table 2:** Nucleotide sequence identities of Irish ECoVs and ECoVs from other continents.

**Nucleotide Identities (%) of Nucleocapsid Gene to:**						
**Name**	**11V11708/IRL**	**13V08313/IRL**	**NC99**	**Obihiro2004**	**Tokachi09**	
**Accession No.**	LC149485	LC149486	EF446615	AB671298	LC061272	
**11V11708/IRL**	-	99.8	98.1	98.6	99.2	
**13V08313/IRL**	99.8	-	98.3	98.7	99.4	
**Name**	**Obihiro12-1**	**Obihiro12-2**	**Hidaka-No.61/2012**	**Hidaka-No.119/2012**		
**Accession No.**	LC061273	LC061274	LC054263	LC054264		
**11V11708/IRL**	99.3	99.4	99.5	99.2		
**13V08313/IRL**	99.6	99.6	99.7	99.4		
**Nucleotide Identities (%) of Spike Gene to:**						
**Name**	**11V11708/IRL**	**13V08313/IRL**	**NC99**	**Obihiro2004**	**Tokachi09**	**Obihiro12-1**
**Accession No.**	LC149487	LC149488	EF446615	AB671299	LC061272	LC061273
**11V11708/IRL**	-	99.5	99.7	98.9	98.6	99.4
**13V08313/IRL**	99.5	-	99.5	98.8	98.5	99.2
**Name**	**Obihiro12-2**	**FRA_2011/1**	**FRA_2011/2**	**FRA_2012/1**	**FRA_2012/2**	**FRA_2012/3**
**Accession No.**	LC061274	KC178705	KC178704	KC178703	KC178702	KC178701
**11V11708/IRL**	99.5	99.5	98.6	99.4	99.5	99.5
**13V08313/IRL**	99.4	99.3	98.4	99.3	99.3	99.3

## Supplementary Materials

The following are available online at https://www.mdpi.com/1999-4915/11/10/946/s1, Figure S1: Alignment of the nucleotide sequences of the region from the p4.7 to p12.7 genes of the ECoV NC99, Tokachi09, Obihiro12-1, Obihiro12-2, 11V11708/IRL/2011 and 13V08313/IRL/2013. Minus signs (−) show missing nucleotides and asterisks (*) show conserved nucleotides.
