# Supplementary material for: The First Detection of Equine Coronavirus in Adult Horses and Foals in Ireland
Source: Viruses. 2019 Oct 14;11(10):946. doi: 10.3390/v11100946 (PMC6832964; doi:10.3390/v11100946)
Supplement: Supplementary file 1 [file viruses-11-00946-s001.pdf]

Figure S1

Alignment of the nucleotide sequences of the region from the p4.7 to p12.7 genes of the ECoV NC99, Tokachi09, Obihiro12-1, Obihiro12-2, 11V11708/IRL/2011 and 13V08313/IRL/2013. Minus signs (−) indicate missing nucleotides and asterisks (*) indicate conserved nucleotides.

p4.7 gene

```
                    ------------------------------------------------------------>
11V11708/IRL/2011   ATGACGATTAATTTCGTCATTT-AACTATTTTATATGA---------------------- 37
13V08313/IRL/2013   ATGACGATTAATTTCGTCATTT-AACTATTTTATATGA---------------------- 37
Obihiro12-1         ATGACGATTAATTTCGTCATTT-AACTATTTTATATGA---------------------- 37
Obihiro12-2         ATGACGATTAATTTCGTCATTT-AACTATTTTATATGA---------------------- 37
NC99                ATGACGATTAATTTCGTCATTTTAACTATTTTATATGACCTGCCTTTGTGGGTTAAACTT 60
Tokachi09           ATGACGATTAATTT---------------------------------------------- 14
                    **************

                    ------------------------------------------------------------>
11V11708/IRL/2011   ----CAACCACTCATGGGTGTACTAATATTAATACTAGTACAGCTCTACATTGGC---TT 90
13V08313/IRL/2013   ----CAACCACTCATGGGTGTACTAATATTAATACTAGTACAGCTCTACATTGGC---TT 90
Obihiro12-1         ----CAACCACTCATGGGTGTAC---TATTAATACTAGTACAGCTCTACATTGGC---TT 87
Obihiro12-2         ----CAACCACTCATGGGTGTAC---TATTAATACTAGTACAGCTCTACATTGGC---TT 87
NC99                GCTACAACCACTCATGGGTGTACTAATATTAATACTAGTACAGCTCTACATTGGCTTATT 120
Tokachi09           ------------------------------------------------------------ 14

                    ->
11V11708/IRL/2011   TAGGCATTTT-------GTTAACACAGCCACCATCGGGTTAAACCGGTTTATGGTGCTAG 143
13V08313/IRL/2013   TAGGCATTTT-------GTTAACACAGCCACCATCGGGTTAAACCGGTTTATGGTGCTAG 143
Obihiro12-1         TAGGCATTTT-------GTTAACACAGCCACCATCGGGTTAAACCGGTTTATGGTGCTAG 140
Obihiro12-2         TAGGCATTTT-------GTTAACACAGCCACCATCGGGTTAAACCGGTTTATGGTGCTAG 140
NC99                TAGGCATTTTTATATGTGTTAACACCACCACCATCGGGTTAAACCGGTTTATGGTGCTAG 180
Tokachi09           ------------------------------------------------------------ 14

11V11708/IRL/2011   TGCTAAATTATATTTTGTTATACTTTATAACTTTAAGCATTTGCTAAAGTTTTTAAGGCC 203
13V08313/IRL/2013   TGCTAAATTATATTTTGTTATACTTTATAACTTTAAGCATTTGCTAAAGTTTTTAAGGCC 203
Obihiro12-1         TGCTAAATTATATTTTGTTATACTTTATAACTTTAAGCATTTGCTAAAGTTTTTAAGGCC 200
Obihiro12-2         TGCTAAATTATATTTTGTTATACTTTATAACTTTAAGCATTTGCTAAAGTTTTTAAGGCC 200
NC99                TGCCAAATTATATTTTGTTATACTTTATAACTTTAAGCAGTTGCTAAAGTTCTTAAGGCC 240
Tokachi09           -------------------ATACTTTATAACTTTAAGCATTTGCTAAAGTTCTTAAGGCC 55
                                       ******************** *********** ********
```

p12.7 gene

```
11V11708/IRL/2011    AACCCTTATTAATGGACATCTGGAGACCTGAGAGGAAATTTCTCCGTTTTACTAATGGAT 263
13V08313/IRL/2013    AACCCTTATTAATGGACATCTGGAGACCTGAGAGGAAATTTCTCCGTTTTACTAACGGAT 263
Obihiro12-1          AACCCTTATTAATGGACATCTGGAGACCTGAGAGGAAATTTCTCCGTTTTACTAACGGAT 260
Obihiro12-2          AACCCTTATTAATGGACATCTGGAGACCTGAGAGGAAATTTCTCCGTTTTACTAACGGAT 260
NC99                 AACCCTTATTAATGGACATCTGGAGACCTGAGAGGAAATTTCTCCGTTTTACTAACGGAT 300
Tokachi09            AACCCTTATTAATGGACATCTGGAGACCTGAGAGGAAATTTCTCCGTTTTACTAACGGAT 115
                     ******************************************************* ****

11V11708/IRL/2011    TTAACGTCCCAGAATTAGAAGATGTCTGTTTTAAATTTAATTACCAATTGCCTAAAGTAG 323
13V08313/IRL/2013    TTAACGTCCCAGAATTAGAAGATGTCTGTTTTAAATTTAATTACCAATTGCCTAAAGTAG 323
Obihiro12-1          TTAACGTCCCAGAATTAGAAGATGTCTGTTTTAAATTTAATTACCAATTGCCTAAAGTAG 320
Obihiro12-2          TTAACGTCCCAGAATTAGAAGATGTCTGTTTTAAATTTAATTACCAATTGCCTAAAGTAG 320
NC99                 TTAACGTCCCAGAATTAGAAGATGTCTGTTTTAAATTTAATTACCAATTTCCTAAAGTAG 360
Tokachi09            TTAATGTCCCAGAATTAGAAGATGTCTGTTTTAAATTTAATTACCAATTTCCTAAAGTAG 175
                     **** ********************************************* **********

11V11708/IRL/2011    GATATTGTAGAGTTCCTAATTATGCTTGGTGTCGTAATCAAGGTAGCTTTTGTGCTACAT 383
13V08313/IRL/2013    GATATTGTAGAGTTCCTAATTATGCTTGGTGTCGTAATCAAGGTAGCTTTTGTGCTACAT 383
Obihiro12-1          GATATTGTAGAGTTCCTAATTATGCTTGGTGTCGTAATCAAGGTAGCTTTTGTGCTACAT 380
Obihiro12-2          GATATTGTAGAGTTCCTAATTATGCTTGGTGTCGTAATCAAGGTAGCTTTTGTGCTACAT 380
NC99                 GATATTGTAGAGTTCCTAATTATGCTTGGTGTCGTAATCAAGGTAGCTTTTGTGCTACAT 420
Tokachi09            GATATTGTAGAGTTCCTAATTATGCTTGGTGTCGTAATCAAGGTAGCTTTTGTGCTACAT 235
                     ************************************************************

11V11708/IRL/2011    TTACCCTTTACGGCAAATCCAAACATTATGATAAATATTTTAGAATAATAACTGGTTTCA 443
13V08313/IRL/2013    TTACCCTTTACGGCAAATCCAAACATTATGATAAATATTTTAGAATAATAACTGGTTTCA 443
Obihiro12-1          TTACCCTTTACGGCAAATCCAAACATTATGATAAATATTTTAGAATAATAACTGGTTTCA 440
Obihiro12-2          TTACCCTTTACGGCAAATCCAAACATTATGATAAATATTTTAGAATAATAACTGGTTTCA 440
NC99                 TCACCCTTTACGGCAAATCCAAACATTATGATAAATATTTTAGAATAATAACTGGTTTCA 480
Tokachi09            TTACCCTTTACGGCAAATCCAAACATTATGATAAATATTTTAGAATAATAACTGGTTTCA 295
                     * **********************************************************
```

```
11V11708/IRL/2011    CAGCGTTCGCCAATACTATAGAGGAGGCTGTTAACAAACTGGTTTTAGAGGCTGTTGATT 503
13V08313/IRL/2013    CAGCGTTCGCCAATACTATAGAGGAGGCTGTTAACAAACTGGTTTTAGAGGCTGTTGATT 503
Obihiro12-1          CAGCGTTCGCCAATACTATAGAGGAGGCTGTTAATAAACTGGTTTTAGAGGCTGTTGATT 500
Obihiro12-2          CAGCGTTCGCCAATACTATAGAGGAGGCTGTTAATAAACTGGTTTTAGAGGCTGTTGATT 500
NC99                 CAGCGTTCGCCAATACTATAGAGGAGGCTGTTAACAAACTGGTTTTAGAGGCTGTTGATT 540
Tokachi09            CAGCGTTCGCTAATACTATAGAGGAGGCTGTTAACAAACTGGTTTTAGAGGCTGTTGATT 355
                     ********** *********************** *************************

11V11708/IRL/2011    TTATTATCTGGCGTAGCCAGAATTTAAATGCTTATGGCTGA                    544
13V08313/IRL/2013    TTATTATCTGGCGTAGCCAGAATTTAAATGCTTATGGCTGA                    544
Obihiro12-1          TTATTATCTGGCGTAGCCAGAATTTAAATGCTTATGGCTGA                    541
Obihiro12-2          TTATTATCTGGCGTAGCCAGAATTTAAATGCTTATGGCTGA                    541
NC99                 TTATTATCTGGCGTAGCCAGAATTTAAATGCTTATGGCTGA                    581
Tokachi09            TTATTATCTGGCGTAGCCAGAATTTAAATGCTTATGGCTGA                    396
                     *****************************************
```
